# Characterization of Hofbauer cell polarization and VEGF localization in human term placenta from active and inactive pregnant individuals

**DOI:** 10.14814/phy2.15741

**Published:** 2023-06-03

**Authors:** Alexandra D. Goudreau, Catherine Everest, Layli Tanara, Velislava Tzaneva, Kristi B. Adamo

**Affiliations:** ^1^ Faculty of Health Sciences University of Ottawa Ottawa Ontario Canada; ^2^ Faculty of Science University of Ottawa Ottawa Ontario Canada

**Keywords:** angiogenesis, Hofbauer cells, macrophage polarization, physical activity, placenta, pregnancy

## Abstract

Physical activity (PA) during pregnancy is associated with parental and fetal health benefits; however, the mechanisms through which these benefits arise are yet to be fully understood. In healthy pregnancies Hofbauer cells (HBCs) comprise a heterogenous population containing CD206^+^ and CD206^−^ phenotypes. In healthy pregnancies, CD206^+^ represent the majority, while dysregulations have been associated with pathological conditions. HBCs have also been identified as potential drivers of angiogenesis. As PA induces changes in macrophage polarization in non‐pregnant populations, this novel study examined the relationship between PA and HBC polarization and to identify which HBC phenotypes express VEGF. Participants were classified as active or inactive, and immunofluorescence cell‐labelling was used to quantify total HBCs, CD206^+^ HBCs, and the proportion of total HBCs expressing CD206. Immunofluorescent colocalization assessed which phenotypes expressed VEGF. Protein and mRNA expression of CD68 and CD206 were measured in term placenta tissue using Western blot and RT‐qPCR, respectively. Both CD206^+^ and CD206^−^ HBCs expressed VEGF. The proportion of CD206^+^ HBCs was elevated in active individuals; however, CD206 protein expression was observed to be lower in active participants. Combined with a lack of significant differences in CD206 mRNA levels, these findings suggest potential PA‐mediated responses in HBC polarization and CD206 translational regulation.

## Graphical abstract

In healthy pregnancies, placental macrophages, Hofbauer cells (HBCs) comprise a heterogenous population containing M1 and M2 phenotypes. This novel study aimed to examine the relationship between PA and HBC polarization and to identify which distinct HBC phenotypes express VEGF. We found that most HBCs were the M2 phenotype and all expressed VEGF.

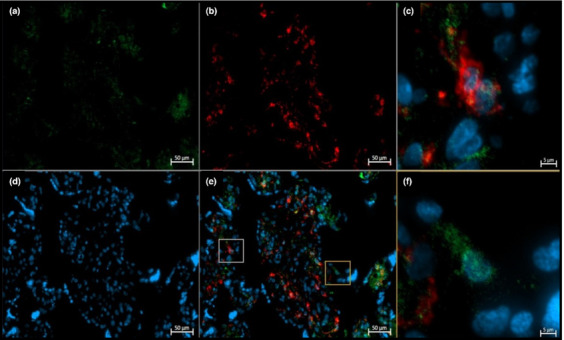

## 1 INTRODUCTION

**FIGURE 1:**
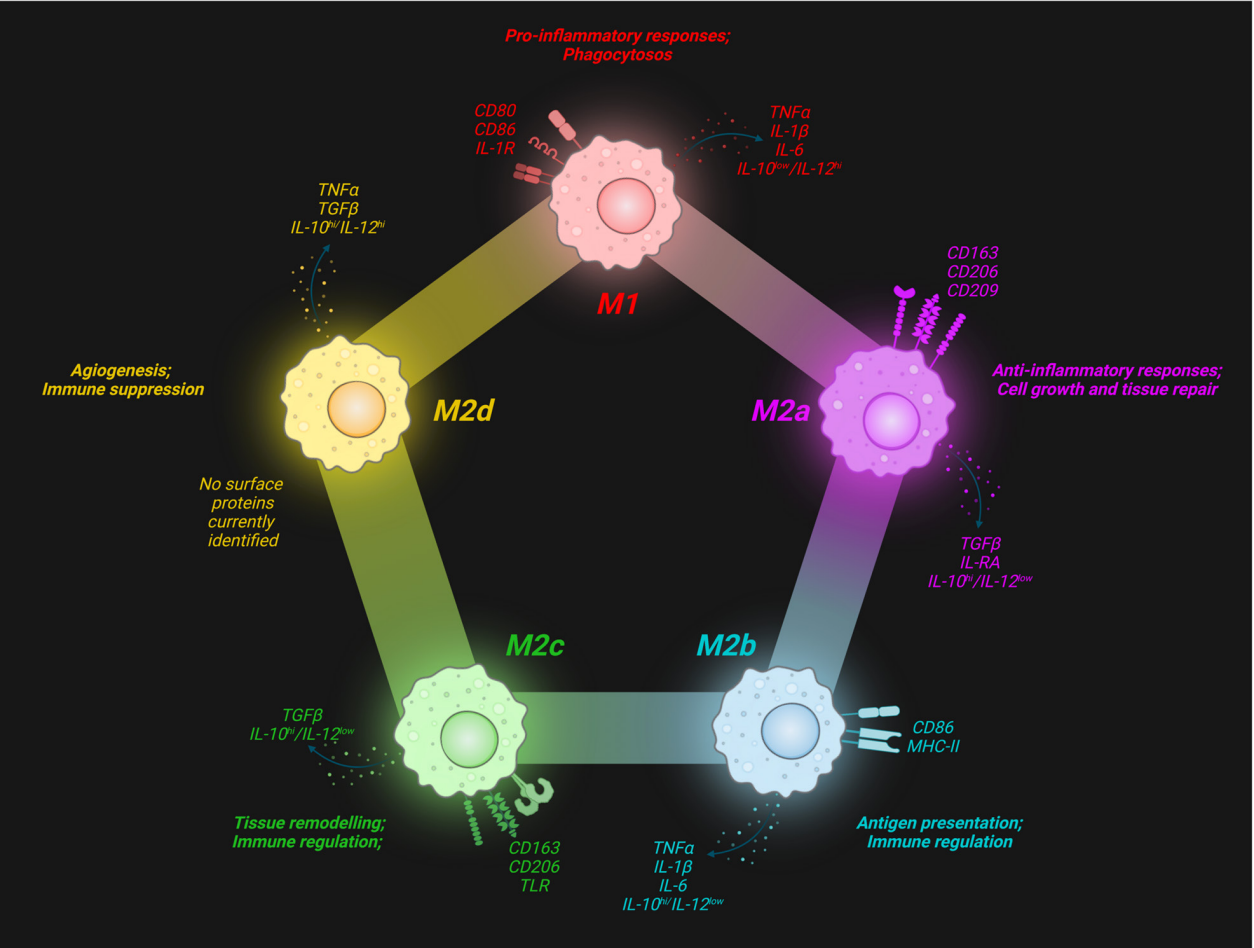
Surface markers and secreted factors of predominant subtypes in the macrophage polarization spectrum. Created with BioRender.com.

Habitual maternal physical activity (PA) reduces the risk of developing pregnancy‐related complications such as gestational diabetes (GDM), hypertension, preeclampsia, and pre‐term birth, while promoting appropriate birth weight (Mottola et al., 2018). As the placenta plays a key part in supporting and maintaining a healthy pregnancy, PA throughout gestation may provide a downstream regulatory influence over fundamental biological processes of placental function. Tissue‐resident macrophages can be identified in most organs throughout the body, and comprise significantly heterogeneous populations to accommodate the different functionalities required in their tissue‐specific niches (Davies et al., 2013). Placenta‐resident macrophages of fetal origin, termed Hofbauer cells (HBCs), perform various functions during pregnancy, including antigen presentation, phagocytosis, and cytokine secretion (Zulu et al., 2019). As is characteristic of macrophages, HBCs possess the ability to polarize between functional states by adapting to their microenvironment (Yao et al., 2019). These phenotypes can generally be divided into M1 and M2 classifications, a pattern characteristic of Type 1 pro‐inflammatory and Type 2 anti‐inflammatory responses, respectively (Rőszer, 2015). Broadly speaking, M1 macrophages are pro‐inflammatory effectors of the immune system, while their anti‐inflammatory M2 counterparts contribute to the regulation and repair of tissues (Zulu et al., 2019). The M2 branch can be further divided into subtypes M2a, M2b, M2c, and M2d (Figure 1). Despite the widespread practice of categorizing macrophages within these discrete subtypes, there is a recognition that it is an oversimplified paradigm. Due to the extensive adaptability and plasticity of macrophages, polarization exists on a continuous spectrum, with a wide range of intermediates that often possess overlapping characteristics (Murray, 2017). Moreover, macrophages develop different functional capabilities and morphologies based on their tissue location (Italiani & Boraschi, 2015). As such, it is important to recognize the pitfalls of strictly classifying macrophages into defined categories with distinct roles.

Bearing in mind the limitations of such classifications, healthy pregnancies are characterized by HBC populations composed of subtypes that most closely resemble M2a, M2b, and M2c phenotypes, with M2a and M2c subtypes comprising the majority of the population (Zhang et al., 2022). The literature clearly indicates that healthy pregnancies are characterized by a lack of M1 HBCs (Reyes & Golos, 2018; Schliefsteiner et al., 2017; Zulu et al., 2019). While M2 macrophages are often generally regarded as anti‐inflammatory, M2b macrophages share several characteristics with their M1 counterparts, including the expression of the surface marker CD86 and secretion of pro‐inflammatory cytokines, such as TNFα, IL‐1β, IL‐6, and IL‐12 (Wang et al., 2019). Moreover, a shift toward a higher proportion of M2b phenotypes has been observed in pregnancies with inflammatory pathologies such as GDM (Schliefsteiner et al., 2017). The cause of this shift has yet to be identified; however, M2b macrophages and their strong immunosuppressive abilities have been linked to the promotion of infections (Wang et al., 2019). The evidence that individuals who develop GDM during their pregnancy are more susceptible to chorioamnionitis, the infection of the placenta and amniotic membranes, lends support to this theory (Bohiltea et al., 2019).

In non‐pregnant human and animal populations, PA has been associated with phenotypic changes in macrophage polarization states (Murray, 2017). Zhang et al. (2020) found that treating macrophages with lactate concentrations corresponding to the physiological levels associated with PA induce a switch from M1 to M2 polarization states (Zhang et al., 2020). Furthermore, M2 tissue‐resident macrophages in non‐placental tissue have been linked to the expression of vascular endothelial growth factor (VEGF) and the promotion of pro‐angiogenic responses. Based on the knowledge of the polarization response of tissue‐resident macrophages to PA and the associated increase in angiogenic properties, it is possible that HBCs provide a similar response in the placenta.

Placental vascularization is necessary to maximize nutrient transfer to the growing fetus and promote proper development. Much like a skeletal muscle responds to PA, an increase in placental parenchymal volume, vascular volume, and villous surface area has been linked to maternal aerobic activity over gestation (Prior et al., 2004). Similar to other tissue‐resident macrophages, such as microglia (Dudiki et al., 2020), HBCs possess angiogenic properties (Loegl et al., 2016; Seval et al., 2007; Zhao et al., 2018). These angiogenic properties include the expression of VEGF, a signaling protein promoting the growth of new blood vessels (Melincovici et al., 2018). While in most macrophage populations, the M2d subtype is the primary driver of VEGF secretion, this phenotype has not been observed in HBCs. The subtype that is the main driver of VEGF production in HBC populations has yet to be identified. A previous study by our lab has shown that as maternal PA increases, so does the protein expression of VEGF and its receptor VEGF receptor‐1 in term placenta (Bhattacharjee, Mohammad, Goudreau, & Adamo, 2021). This finding suggests that PA over gestation may enhance placental pro‐angiogenic factors, promoting placental vascularization.

The primary aim of this preliminary study was to investigate the effect of PA throughout pregnancy on HBC presence and polarization in previously banked tissue samples from term placenta. Given that the previously published research by Bhattacharjee, Mohammad, and Adamo (2021) and Bhattacharjee, Mohammad, Goudreau, and Adamo (2021) illustrated a higher level of VEGF expression in the term placenta of active participants, we also endeavored to examine which HBC phenotypes expressed the angiogenic factor VEGF.

## 2 METHODS

### 2.1 Ethics statement

Participants provided informed written consent to participate in the PhysicaL Activity and diEtary implicatioNs Throughout pregnAncy (PLACENTA) study, at the University of Ottawa. The PLACENTA study was approved by the Research Ethics Board (REB) of the University of Ottawa (file number: H11‐15‐29) and conformed to all aspects of the Declaration of Helsinki.

### 2.2 Participant recruitment

Participants were recruited as a part of the PLACENTA study from the Ottawa region (Ottawa, Ontario) via flyers posted at health clinics, community centers, universities, and social media. Interested individuals were pre‐screened by phone to assess their eligibility. The inclusion criteria for the pregnant individuals were as follows: 18–40 years old; able to communicate in either English or French; less than 28 weeks gestation; weight stable for at least six months before conception (±2 kg); carrying a singleton fetus; and having a self‐reported pre‐pregnancy body mass index (BMI) of normal or overweight (18.5–29.9 kg/m^2^). Exclusion criteria for study recruitment were having contraindications to exercise; being diagnosed with pre‐pregnancy diabetes; or having untreated thyroid disease. Individuals participated in an in‐lab visit twice during their pregnancy; once during mid‐gestation (between 24 and 28 weeks of gestation) and once during late gestation (between 34 and 38 weeks).

### 2.3 Physical activity analysis

After completing the assessments at both gestational timepoints, participants were given a take‐home package that included an omnidirectional Actical® accelerometer (Philips Respironics). Participants were instructed to wear the accelerometer around their waist for waking hours over seven consecutive days to record periods of free‐living PA. To be included in further analyses, participants were required to have three valid days with a minimum wear time of ten hours per day (da Silva et al., 2021). Data analyses were performed as previously described (Tremblay & Connor Gorber, 2007) using SAS version 9.4 as per the Canadian Health Measures Survey procedures. Data output from the accelerometers were used to measure the minutes of PA accumulated during the week. Participants were classified as active or inactive based on the evidence‐based 2019 Canadian guideline for physical activity during pregnancy recommendation that pregnant individuals should perform at least 150 min of moderate PA a week, or an average of 21.4 min per day (Mottola et al., 2018). Individuals who averaged 21.4 min per day of moderate‐to‐vigorous PA (MVPA) or more at both mid and late gestation were classified as active. Conversely, participants that did not reach this average at both gestational time points were classified as inactive.

### 2.4 Sample collection

Term placentae were sampled within an hour of delivery to preserve specimen integrity. Large tissue biopsies (approximately 2.5 cm^3^) were dissected from central and peripheral cotyledons. Tissue samples were further dissected into small pieces (~1 cm^3^), then placed in cryovials and flash‐frozen in liquid nitrogen. Frozen placenta tissue was powdered on ice and homogenized in radioimmunoprecipitation assay (RIPA) buffer (BioRad**)** using Powergen 125 homogenizer (Fisherbrand). The protein lysate was centrifuged at 1000 *g* for 10 min at 4°C and stored at −80°C until further analysis. Full‐thickness histological samples were taken from healthy cotyledons of the placenta, avoiding areas of necrosis and calcification. Sections were rinsed in non‐sterile phosphate‐buffered saline (PBS). For formalin‐fixed, paraffin‐embedded samples (FFPE), the dissected sections were placed in 10% formalin for 48 h, then rinsed three times in non‐sterile PBS and subsequently preserved in paraffin blocks. Additional sections were placed into plastic cryomolds containing O.C.T embedding compound. Cryomolds were wrapped in aluminum foil and flash‐frozen in liquid nitrogen.

### 2.5 Western blotting

Twenty to forty μg of total placental protein were loaded on Mini‐PROTEAN® TGX gel (Bio‐Rad) and resolved at 150 volts for 1 h. The proteins were transferred onto a polyvinylidene difluoride (PVDF) membrane (Bio‐Rad) and blocked with 5% powdered milk in tris buffered saline solution with 0.05% tween‐20 (TBST) for 1 h at room temperature (RT). Membranes were incubated overnight at 4°C with mouse monoclonal anti‐CD68 (1:500, Abcam Cat# ab201973, RRID:AB_2936513) and rabbit polyclonal anti‐CD206 (1:800, Abcam Cat# ab64693, RRID:AB_1523910). The following day, blots were washed with TBST and incubated with 1:5000 dilutions of horse radish‐peroxidase conjugated secondary antibodies (Goat Anti‐Mouse IgG Bio‐Rad Cat# 1706516, RRID:AB_2921252; Goat Anti‐Rabbit IgG Bio‐Rad Cat# 1706515, RRID:AB_2617112) for 1 h at RT. The blots were developed using Clarity ECL Western Substrate (Bio‐Rad) and imaged using ChemiDoc™ XRS+ Imaging System (Bio‐Rad). Membranes were permanently stained with 1% Amido Black for total protein lane quantification. Band expression and total protein expression were analyzed by densitometry (ImageJ, Bio‐Rad). Placental protein expression for CD68 and CD206 was standardized to total participant pooled protein lysate samples.

### 2.6 Immunofluorescence

**FIGURE 2:**
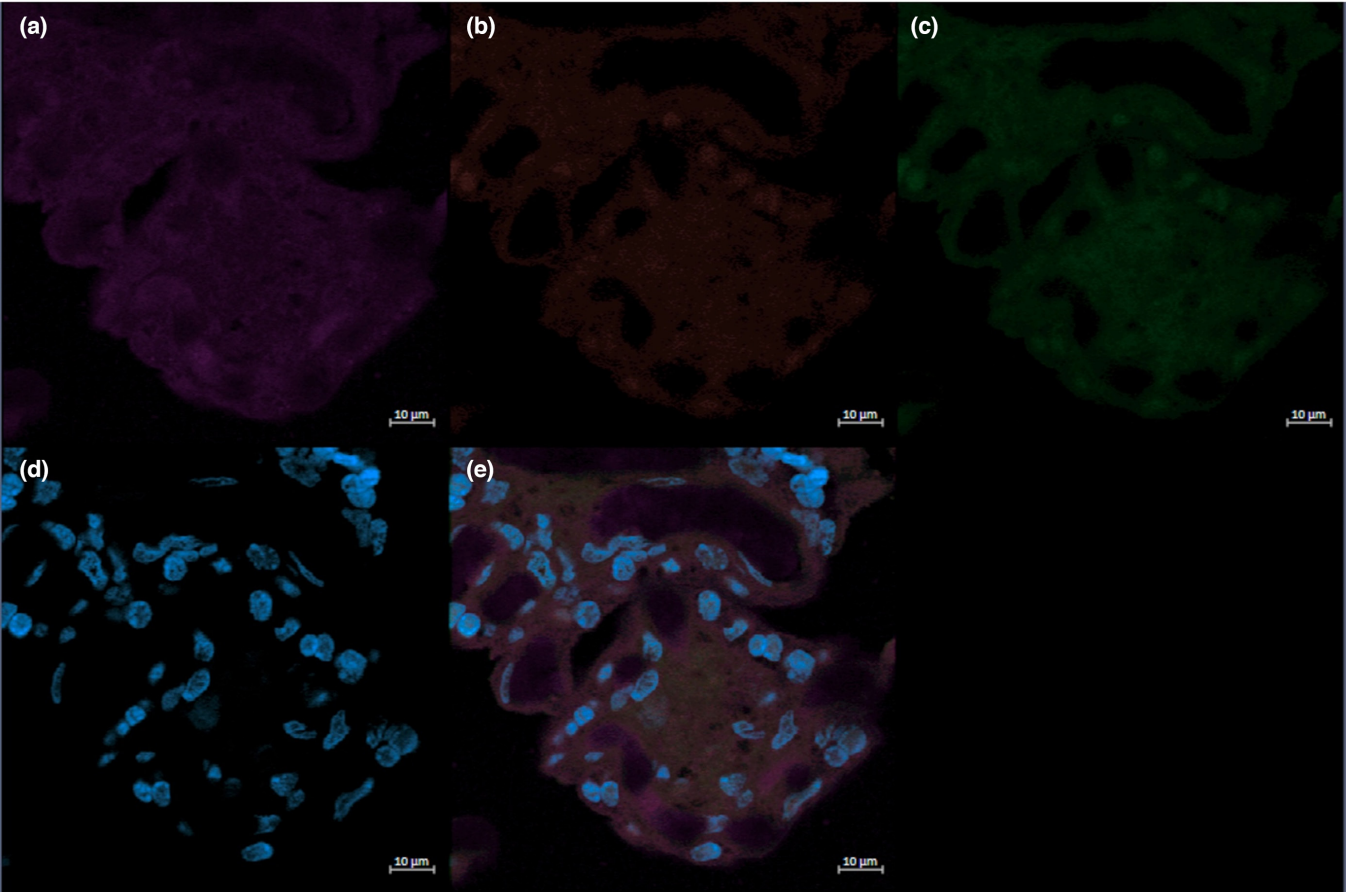
The negative control for immunofluorescent staining of VEGF (a, purple), CD206 (b, red), CD68 (c, green), and DAPI (d, blue) and the corresponding merged image (e) is shown.

Placental FFPE tissue was processed into slides by the Louise Pelletier Histology Core at the University of Ottawa. Tissue was sectioned at 4 μm thickness for analysis. Slides were then deparaffinized and rehydrated using xylene and a graded series of ethanol dilutions before being rinsed in double‐distilled water. Heat‐mediated antigen retrieval was performed using sodium citrate buffer (10 mM, pH 6.0). Tissue sections were permeabilized in 0.2% Triton‐X in TBS for 20 min at RT. Sections were then incubated in 10% bovine serum albumin (BSA) for 1 h at RT to block non‐specific binding, then incubated in 0.1% Sudan Black in 75% EtOH (wt/vol) for 10 min at RT to quench tissue autofluorescence. The primary antibodies for CD68 (mouse monoclonal Abcam Cat# ab201973, RRID:AB_2936513) and CD206 (rabbit polyclonal Abcam Cat# ab64693, RRID:AB_1523910) were diluted in TBST at 4 and 2 μg/mL, respectively, before being applied to the sections, and then incubated overnight at 4°C. This step was omitted in negative controls (Figure 2). Following the application of the primary antibodies, slides were washed three times in TBST, then incubated in 1:250 and 1:1000 dilutions of Alexa 488 goat anti‐mouse secondary antibody (Thermo Fisher Scientific Cat# A28175, RRID:AB_2536161), and Alexa 594 goat anti‐rabbit secondary antibody (Thermo Fisher Scientific Cat# A‐11012, RRID:AB_2534079) respectively for 1 h at RT in the dark. Slides were washed three times for five minutes each in TBST in the dark, then mounted with Prolong Gold with DAPI. Edges were sealed with nail polish.

Immunofluorescence was also used to localize VEGF in relation to HBCs. Frozen histological tissue samples were brought to room temperature and rinsed in PBS, then fixed in 4% paraformaldehyde at RT for 5 min. After being washed in PBST (0.2% Triton‐X), sections were blocked in 10% BSA in PBST for 1 h and quenched with 0.1% Sudan Black in 75% EtOH for 10 min at RT. CD68 (mouse monoclonal Abcam Cat# ab201973, RRID:AB_2936513) and CD206 (rabbit polyclonal Abcam Cat# ab64693, RRID:AB_1523910) primary antibodies were diluted in PBST at 4 and 2 μg/mL, respectively. On day one, sections were incubated in the diluted primary antibodies overnight at 4°C. On day two, slides were washed of primary antibodies thoroughly with PBST and the secondary antibodies were applied (Thermo Fisher Scientific Cat# A28175, RRID:AB_2536161; Thermo Fisher Scientific Cat# A‐11012, RRID:AB_2534079) at 1:1000 dilution for 1 h at RT. After thoroughly washing the secondary antibodies 3 × 5 min with TBST, the anti‐VEGF primary antibody (mouse monoclonal; Novus Cat# NB100‐664, RRID:AB_10001947) was diluted at 1:50 in TBST and applied to the slides overnight at 4°C. On day three, slides were washed three times in TBST and incubated in a 1:500 dilution of Alexa 647 goat anti‐mouse secondary antibody (Thermo Fisher Scientific Cat# A32728, RRID:AB_2633277) for 1 h at RT. This three day protocol ensured that anti mouse secondary antibodies recognized their primary antibody targets. Slides were then washed and mounted as described above.

### 2.7 Cell quantification and analysis

Slides were imaged using a fluorescent microscope (Zeiss AxioObserver M2) equipped with blue (excitation 390/22 nm, emission 460/50 nm), green (excitation 470/40 nm, emission 525/50 nm) and red (excitation 560/40 nm, emission 630/75 nm) filters to visualize the cells of interest. Eight representative images of each section from all 22 participant samples were acquired to count the CD68 and CD206 positive cells. Bearing in mind the limitations of classifying HBCs into discrete categories, as well as the M2b‐like characteristic of secreting pro‐inflammatory cytokines, HBCs were identified as either CD68^+^CD206^+^ (indicative of an M2a‐ or M2c‐like state) or CD68^−^CD206^−^ (indicative of an M2b‐like state). The number of cells stained with the pan‐macrophage marker CD68 represented total number of Hofbauer cells. Cell numbers were normalized to the fraction of tissue coverage (FTC) in the image using Image J (NIH). To do so, images were converted to a binary color scheme where black pixels represented tissue and white pixels represented intervillous space. Black pixels were quantified by the program and divided by the number of total pixels to obtain the FTC. Cell counts were divided by the FTC to calculate the adjusted values, which were used for statistical analyses.

### 2.8 RNA isolation and quantitative real‐time polymerase chain reaction

Total RNA was isolated from term placenta tissue as previously described (Bhattacharjee, Mohammad, Goudreau, & Adamo, 2021). From the isolated RNA, 1 μg was reverse transcribed into cDNA using iScript™ cDNA Synthesis Kit (1708891; Bio‐Rad Laboratories). The Roto‐Gene RG‐3000 system (Corbett Research) was used to amplify cDNA through a real‐time quantitative polymerase chain reaction (qPCR). Predesigned qPCR 20X probes for CD68 (Hs.PT.58.2488447) and CD206 (Hs.PT.58.15093573) were purchased from Integrated DNA Technologies. For all samples, YWHAZ (Hs.PT.39a.22214858) was used as an endogenous control (Meller et al., 2005). Threshold cycle (TC) values were obtained for each sample, and used to analyze the relative gene expression via the 2^−ΔΔCT^ method described by Livak and Schmittgen (2001). Gene expression values were normalized to the endogenous control, and the relative gene expression of physically active participants was determined in comparison to the gene expression of their physically inactive counterparts.

### 2.9 Statistical analysis

All data are presented as mean ± standard deviation. The gene expression analysis software Rotor‐Gene 6 (version 6.1; Corbett Research) was used to analyze qPCR data and GraphPad Prism software (version 9.0.0, GraphPad Software Inc.) was used for all statistical analyses. The Shapiro–Wilks test was used to assess normality, and the ROUT method employed to test for outliers. Unpaired *t*‐tests or Mann–Whitney *U* tests, where appropriate, were used to assess the statistical difference of participant demographics, Western blot, immunofluorescence, and qPCR data between active and inactive participants. Pearson correlations were also used to analyze the number of CD68^+^ and CD68^+^CD206^+^ cells in comparison to the average minutes of MVPA/day obtained by each participant. Statistical significance was defined as *p* ≤ 0.05.

## 3 RESULTS

### 3.1 Participant demographics

Maternal demographic information and newborn outcomes are described in Table 1 according to PA status throughout gestation. By study design, physically active participants had significantly higher MVPA (min/day) than their inactive counterparts in both mid‐ and late‐ gestation (*p* < 0.001). Maternal age, height, pre‐pregnancy weight, or pre‐pregnancy BMI did not differ significantly between active and inactive groups. Similarly, there were no significant differences in newborn birth weight or birth length between physically active and inactive individuals.

**TABLE 1:** Study participant maternal demographics and newborn outcomes (*n* = 21).

	Active (*n* = 11)	Inactive (*n* = 10)	*p*‐Value
Maternal demographics			
Maternal age (years)	32.45 ± 2.98	32.90 ± 2.69	0.7239
Height (cm)	165.37 ± 6.57	168.10 ± 7.26	0.3775
Pre‐pregnancy weight (kg)	64.25 ± 10.33	67.57 ± 13.28	0.5275
Pre‐pregnancy BMI (kg/m^2^)	23.45 ± 2.67	23.66 ± 3.00	0.8641
Gestational age at birth (weeks)	41.27 ± 3.08	40.54 ± 1.04	>0.9999
Mid gestation MVPA (min/day)	46.03 ± 10.03	6.03 ± 3.96	<0.0001
Late gestation MVPA (min/day)	34.27 ± 9.10	3.7 ± 3.54	<0.0001
Newborn outcomes			
Birth weight (kg)	3.28 ± 0.38	3.58 ± 0.43	0.1075
Birth length (cm)	49.85 ± 2.52	51.78 ± 1.98	0.0672
Sex, *n*			
Male	7	4	
Female	4	6	

### 3.2 Placental CD206 expression is significantly higher in physically inactive individuals

**FIGURE 3:**
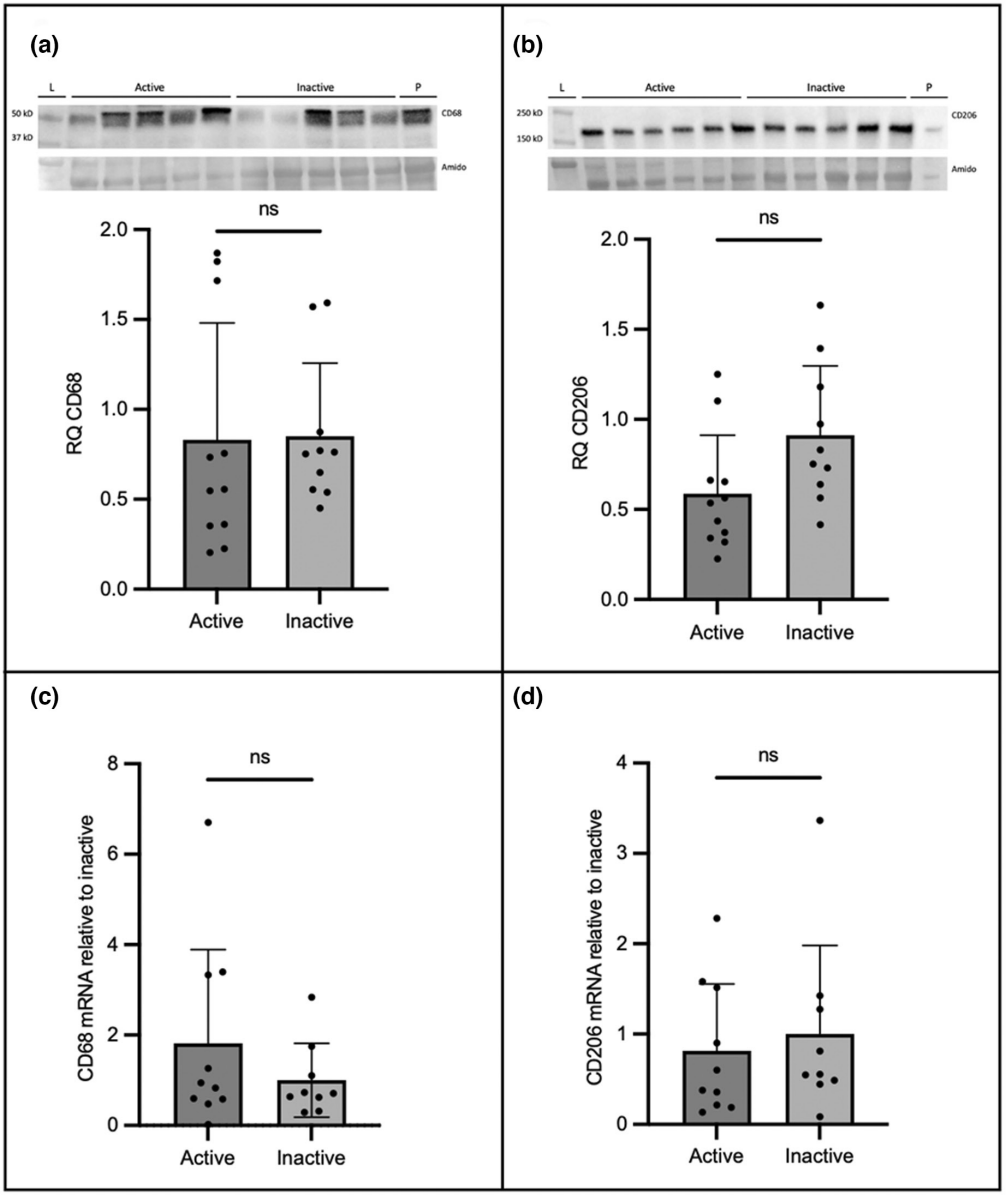
Representative immunoblots and the corresponding semi‐quantitative densitometric analysis are shown for CD68 (a) and CD206 (b). The relative quantification of mRNA for CD68 and CD206 are illustrated in panels (c) and (d), respectively. All data are represented as mean ± SD. *p < 0.05.

Placental CD68 and CD206 protein expression were analyzed to determine the difference in protein levels between active and inactive participants by parametric and non‐parametric *t*‐test, where applicable. No significant differences in the expression of CD68 were found between active and inactive individuals (*p* > 0.05); however, the protein expression of CD206 was lower in active pregnancies compared to inactive (*p* = 0.050; Figure 3a,b).

### 3.3 CD206 and CD68 mRNA expression did not differ based on activity status

The mRNA levels of CD206 and CD68 (Figure 3c,d) in the placenta was investigated by qPCR using RNA isolated from term placental tissue using non‐parametric *t*‐tests. No significant differences in CD206 (*p* = 0.720) or CD68 (*p* = 0.604) mRNA were observed.

### 3.4 Physically active individuals have higher proportions of M2 HBCs

**FIGURE 4:**
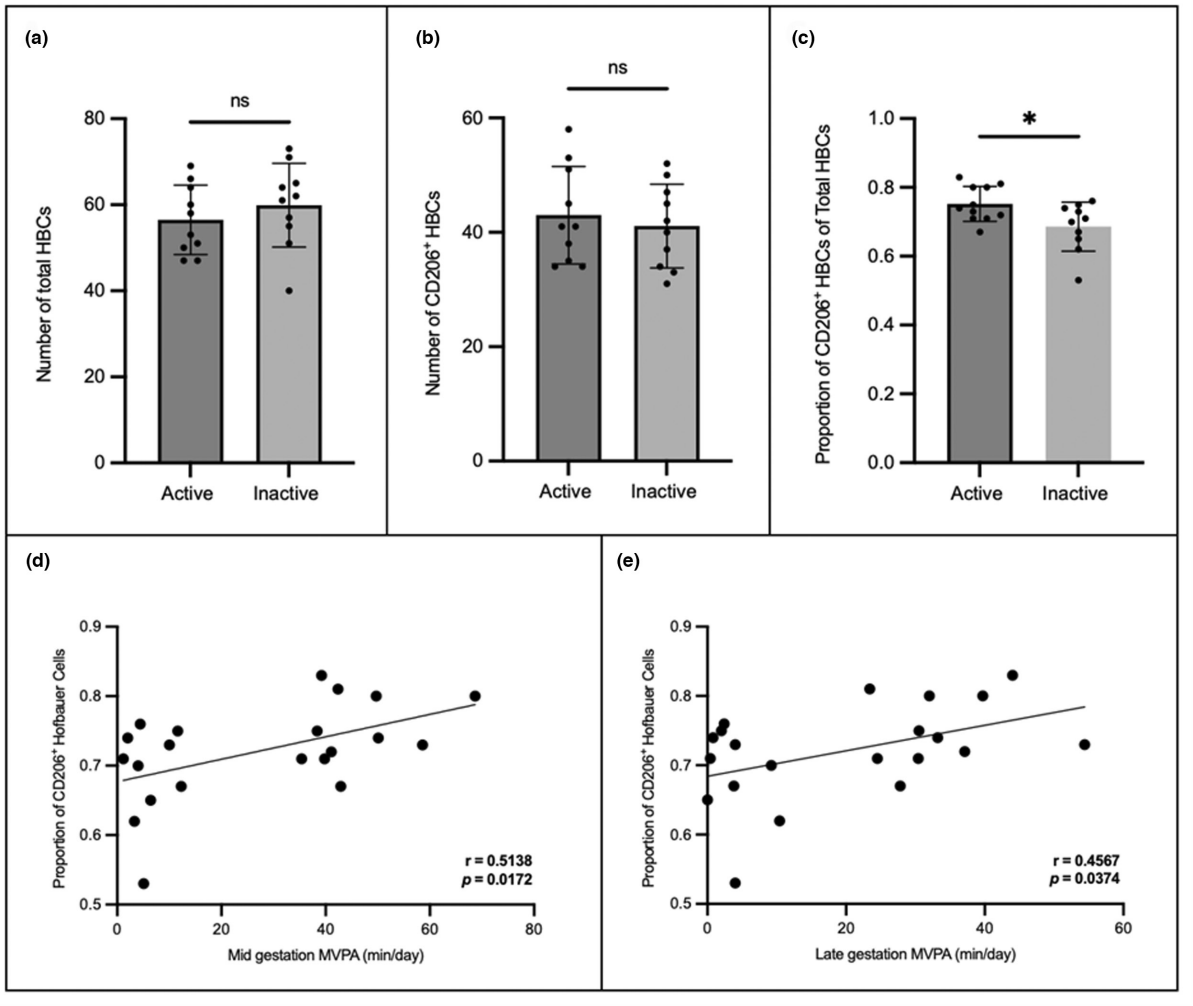
The (a) number of total Hofbauer cells and (b) number of CD206+ Hofbauer cells did not significantly differ between active and inactive individuals; however, the proportion of CD206+ Hofbauer cells (c) was significantly higher in active participants. a–c data are represented as mean ± SD. There were significant correlations between the proportion of CD206+HBCs and average minutes of moderate to vigorous physical activity (MVPA) in mid (d) and late (e) gestation. *p < 0.05.

**FIGURE 5:**
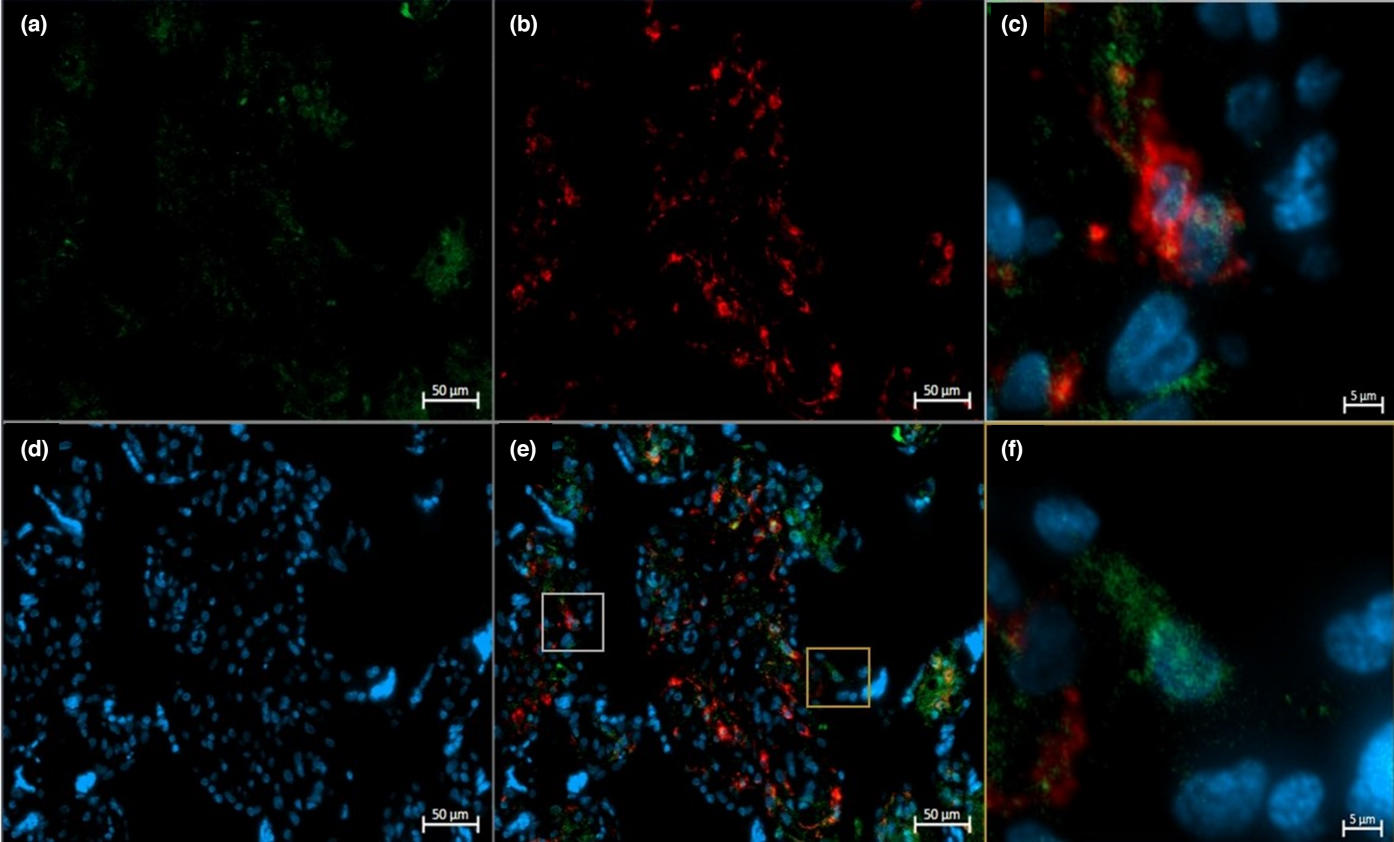
Representative immunofluorescent staining of CD68 (a, green), CD206 (b, red), and DAPI (d, blue) in the term placenta of an active participant. The yellow box highlights a CD206+ (M2a/M2c) Hofbauer cells, while the gray box highlights a CD206− (M2b) Hofbauer cells on the merged (e) image. Magnified images of CD206+ (c) and CD206− (f) Hofbauer cells are shown.

**FIGURE 6:**
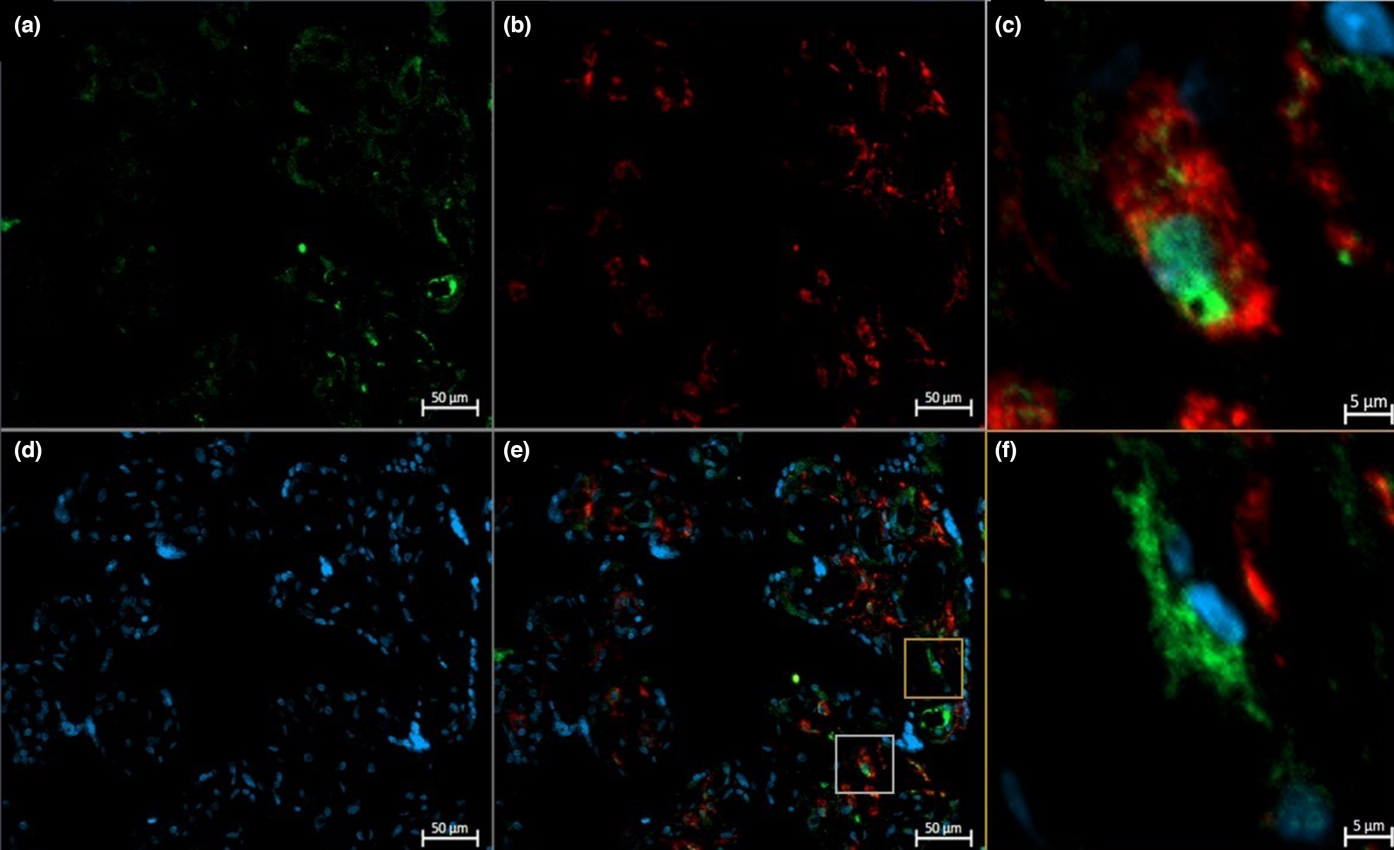
Representative immunofluorescent staining of CD68 (a, green), CD206 (b, red), and DAPI (d, blue) in the term placenta of an inactive participant. The yellow box highlights a CD206+ (M2a/M2c) Hofbauer cells, while the gray box highlights a CD206− (M2b) Hofbauer cells on the merged (e) image. Magnified images of CD206+ (c) and CD206− (f) Hofbauer cells are shown.

Immunofluorescence studies identified total HBCs (CD68^+^) and CD68^+^CD206^+^ HBCs in the term placenta of active and inactive participants (Figures 4, 5, 6). Total HBCs and CD206^+^ HBCs were counted to determine the differences in polarization between groups. Using the ROUT method, one outlier in the active group of both the absolute numbers of total HBCs and CD206^+^ HBCs data was identified and excluded from further analysis. No significant differences were found in the absolute number of total HBCs (*p* = 0.405) and CD206^+^ HBCs (*p* = 0.600). However, the number of CD206^+^ HBCs as a proportion of the total was significantly higher in physically active individuals (*p* = 0.024). There was a significant correlation between the proportion of CD206^+^ HBCs and the average minutes of MVPA achieved per day in both the mid (*r* = 0.514, *p* = 0.017) and late (*r* = 0.457, *p* = 0.037) gestation, with large and medium effect sizes, respectively. No significant correlations between the absolute numbers of total HBCs or CD206^+^ HBCs and MVPA (min/day) were observed at either timepoint (data not shown).

### 3.5 Colocalization of VEGF and HBC markers

**FIGURE 7:**
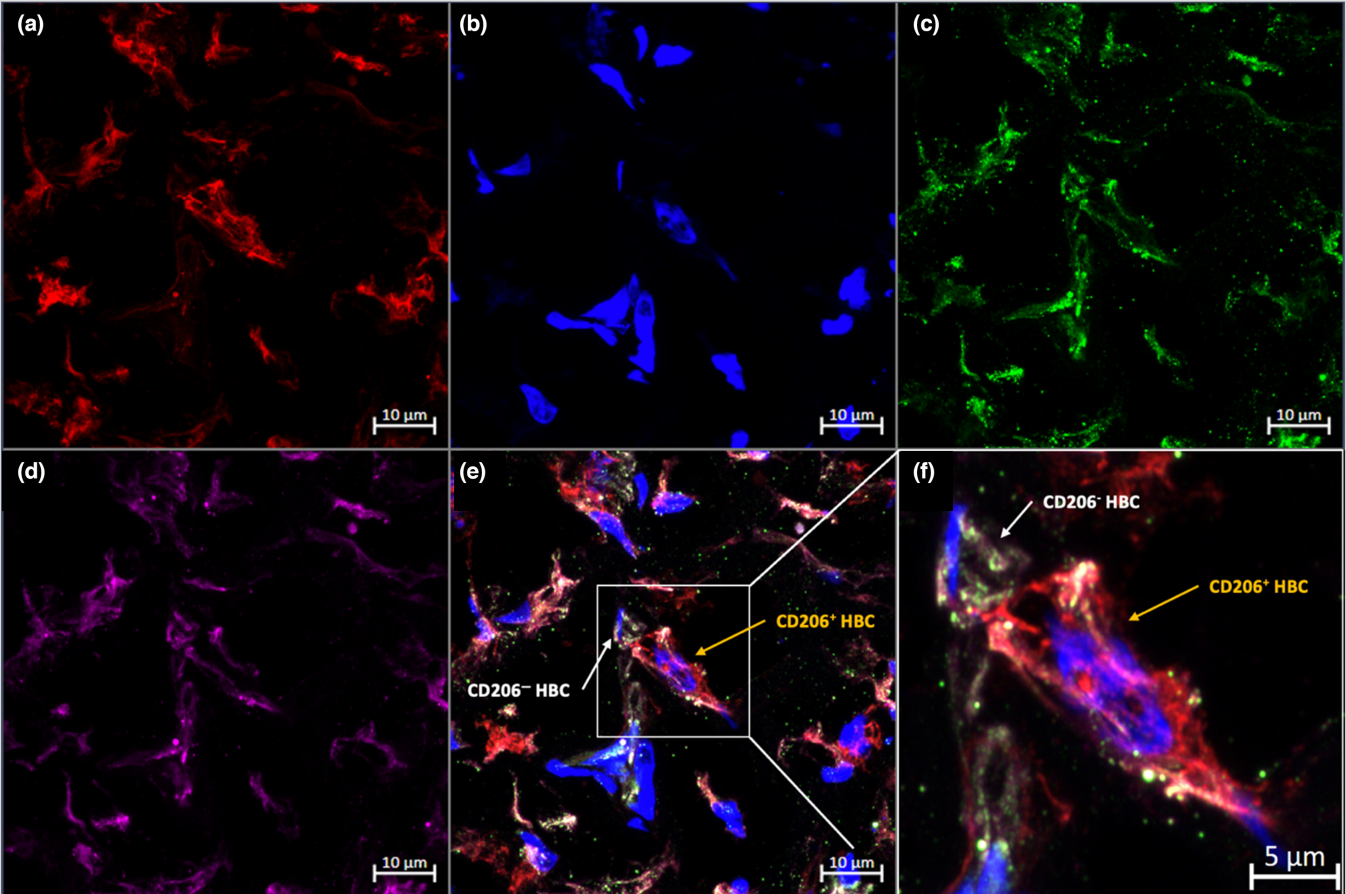
Representative immunofluorescent staining of CD206 (a, red), DAPI (b, blue), CD68 (c, green), and VEGF (d, purple) in term placenta. The white box highlights a CD206+ (M2a/M2c) Hofbauer cells and a CD206− (M2b) Hofbauer cells on the merged (e) image. Magnified images of CD206+ (f) Hofbauer cells are shown.

Figure 7 displays images from the immunofluorescence studies examining the localization of VEGF, the pan‐macrophage marker CD68, and M2a/M2c macrophage marker CD206. In term placenta, VEGF was colocalized within cells expressing both CD68 and CD206, indicative of an M2a‐ or M2c‐like phenotype. Interestingly, VEGF was also found in cells that only expressed CD68, indicative of an M2b‐like phenotype.

## 4 DISCUSSION

Previous research has shown that PA can polarize macrophages from a pro‐inflammatory to an anti‐inflammatory state in circulation, as well as in adipose, lung, and central nervous system tissues (Blanks et al., 2020; Mee‐Inta et al., 2019; Rentz et al., 2020; Shi et al., 2020; Silveira et al., 2016). However, this study is the first to explore the association between gestational PA and the polarization of HBCs. While there were no differences in the absolute numbers of CD206^+^ HBCs or total HBCs between physically inactive and active participants, active individuals had significantly higher levels of CD206^+^ HBCs as a proportion of the total. Due to variations in the intervillous space, absolute cell count numbers were divided by the percentage of tissue coverage in the image. By doing so, the risk of observing differences in cell counts attributable to differences in tissue coverage was mitigated. In observing significant differences in the CD206^+^ HBCs as a proportion of the total HBCs only, it is possible that looking at absolute numbers of polarized and total HBCs is an insufficient method of analysis. Additionally, there were large and significant correlations between the proportion of CD206^+^ HBCs and minutes of MVPA per day in both mid and late gestation. Our findings suggest that PA engagement in pregnancy is a potential determinant of HBC polarization during pregnancy.

The function of the pan‐macrophage marker CD68 has not been extensively investigated; however, it has been shown that the protein can be shuttled to the cell surface, where it is involved in the binding of oxidized low‐density lipoprotein (OxLDL; Chistiakov et al., 2017). The ability of CD68 to bind to OxLDL may play a role in the development of foam cells, which have been observed in preeclampsia. No differences were found in the number of CD68^+^ cells, CD68 protein expression, or mRNA levels, indicating that PA likely does not influence the expression CD68 in term placenta.

CD206 is expressed predominantly by macrophages and monocytes, along with dendritic, lymphatic, and endothelial cells (Azad et al., 2014). Upregulation of CD206 has been observed in a multitude of conditions, including cancers and inflammatory diseases (Choi et al., 2017; Nielsen et al., 2020). Tumor‐associated macrophages (TAMs), thought to contribute to the progression of cancer through immunosuppression and matrix remodeling, have enhanced levels of CD206 (Choi et al., 2017; Martinezpomares, 2001; Nielsen et al., 2020). Elevated expression of CD206 contributes to mannose‐dependent endocytosis, as well as the uptake of collagen, contributing to matrix deposition, fibrosis, and inflammation (Arlt et al., 2020; Choi et al., 2017; Nielsen et al., 2020). In the current study, while the proportion of CD206^+^ HBCs was statistically higher in active individuals, the protein expression of CD206 was significantly higher in inactive individuals. It is possible that other cells expressing CD206, such as endothelial cells, may be responsible for the higher levels of placental CD206 protein observed in those characterized as inactive. However, acute bouts of PA are thought to result in a temporary hypoxic state in the placenta; such conditions have been shown to promote endothelial cell proliferation (Bhattacharjee, Mohammad, & Adamo, 2021; Wong et al., 2017). This proliferation would result in higher CD206 levels, which is opposite to what our study observed. Additionally, to the best of our knowledge, there is no literature suggesting that PA, hypoxia, or inflammation differentially regulates the expression of CD206 within placental endothelial cells. Therefore, it is improbable that the higher level of CD206 observed in inactive individuals is due to endothelial cells; for these reasons, we posit that the difference in CD206 is due to an HBC response. As a significant difference was observed between active and inactive participants in placental CD206 protein expression, but not in mRNA levels, the potential PA‐mediated downregulation of CD206 likely occurs during translation. Therefore, we posit that PA may downregulate placental CD206 expression at a translational level in HBCs, potentially leading to decreased inflammation and fibrosis. This hypothesis, as well as the effects of PA‐mediated changes in CD206, need to be explored in future research and functional studies.

The literature shows that in uncomplicated pregnancies, HBCs do not express an M1 phenotype (Schliefsteiner et al., 2017). Due to this observation, it is plausible that cells identified as CD206^−^ HBCs in the current study possessed an M2b phenotype. Interestingly, while M2b macrophages have been demonstrated to share characteristics with M1 macrophages, including the secretion of TNF‐α, IL‐1β and IL‐6, they also possess regulatory characteristics and are involved in immunosuppression. As mentioned previously, an increase in M2b phenotypes has been associated with GDM (Schliefsteiner et al., 2017). While, to the best of our knowledge, there is no current literature describing the role of M2b macrophages in pregnancies complicated by infections or conditions such as preeclampsia, M2b macrophages in other tissues can exacerbate bacterial, viral, and fungal infections by blunting the immune response (Wang et al., 2019). Furthermore, cytokines secreted by the M2b phenotype, including TNF‐α, IL‐1β, and IL‐6 have been associated with preeclampsia. Based on the current knowledge, increased presence of M2b phenotypes may contribute to the development of preeclampsia. While it has been demonstrated that healthy pregnancies are characterized by a lack of M1 HBCs, the expression of these cytokines can also be attributable to the M1 phenotype. Przybyl et al. discovered that in the presence of preeclampsia, HBCs downregulate the expression of CD74, a human leukocyte antigen class II histocompatibility antigen‐γ chain (Przybyl et al., 2016). The Przybyl team posited that this downregulation induces a phenotypic switch in HBCs from an M2 to an M1 state (Przybyl et al., 2016). HBC populations in pregnancies complicated by pathologies should be studied to determine whether complications induce an M1 phenotype, as well as the potential effects this would have on the expression of secreted factors, including cytokines.

As a previous study by our lab identified that physically active individuals had an increased level of VEGF expression when compared to inactive individuals (Bhattacharjee, Mohammad, Goudreau, & Adamo, 2021), we aimed to investigate if this difference related to expression within different HBC subtypes. In examining the colocalization of VEGF and CD206, VEGF was distinctly localized with both CD206^+^ HBCs and CD206^−^ HBCs. This specific localization indicates that the potential angiogenic action of HBCs is likely not restricted to M2a‐ and M2c‐like phenotypes but is a characteristic of all M2 HBCs in healthy pregnancies. Future research should utilize fluorescence‐activated cell sorting (FACS) to isolate subtypes of HBCs, including M1, M2a, M2b, and M2c, as well as enzyme‐linked immunosorbent assays (ELISA) to determine which phenotype is the main driver of VEGF production in both healthy and complicated pregnancies.

**FIGURE 8:**
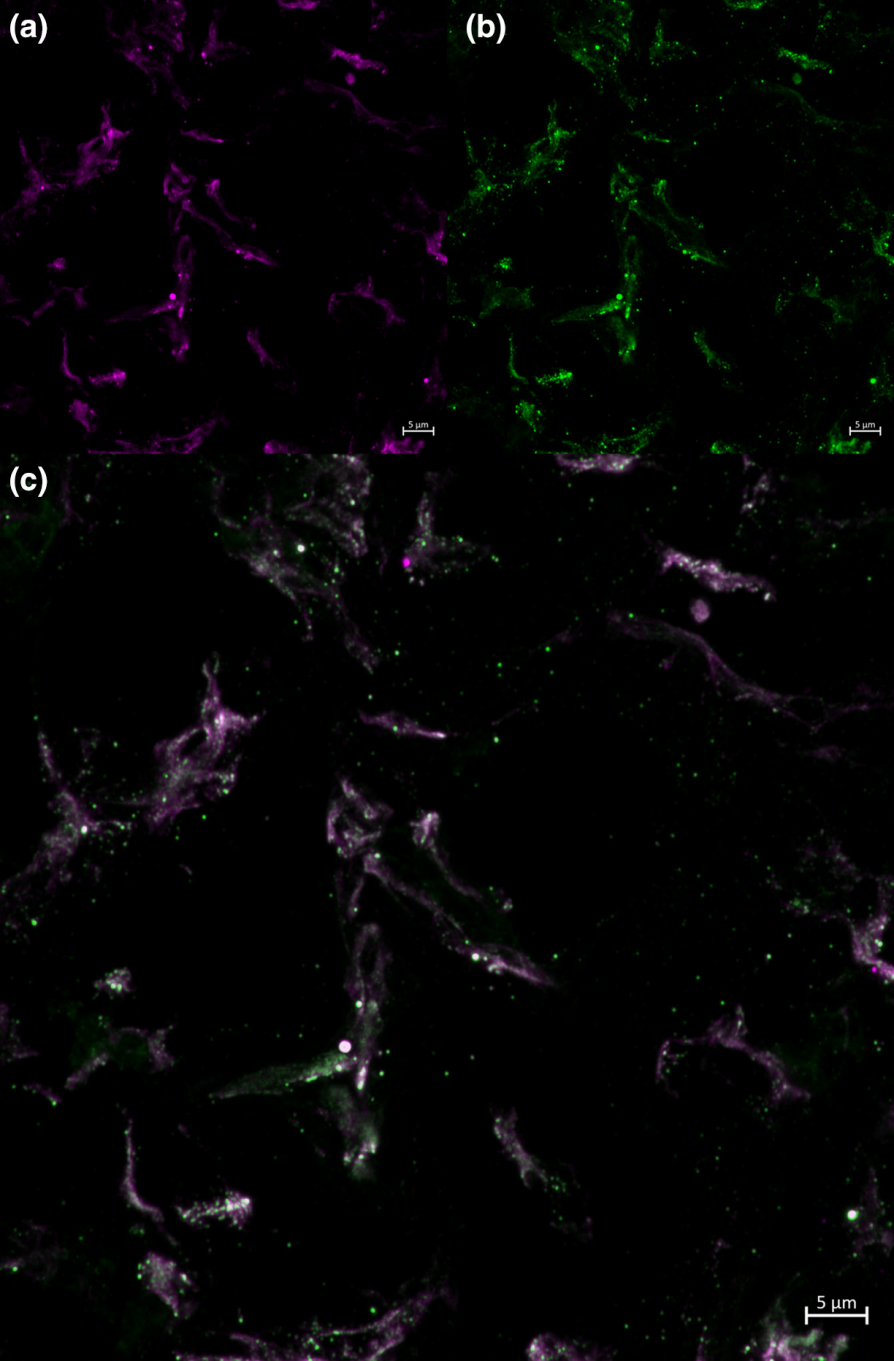
This image illustrates the distinct staining pattern of CD68 and VEGF of Hofbauer Cells with mouse anti CD68 and mouse anti VEGF using 3‐day sequential staining protocol. (a) mouse anti VEGF, (b) mouse anti CD68, (c) merged digitally magnified images. Panel c demonstrates distinct patterns of staining for VEGF and CD68 within cells. This is a maximum projection from a z stack of Hofbauer cells captured using Zeiss LSM880 with AirScan Processing confocal microscope at 20× magnification. Scale bar is 5 μm.

It is important to note that inherent limitations are associated with the current study. The sample size is small and relatively homogenous; therefore, it is possible that results may not be representative of the population. As this investigation was carried out with frozen, banked tissue samples, experiments requiring fresh tissue, such as the isolation of primary HBC cultures to identify HBC polarization with flow cytometry, could not be conducted. Due to a lack of non‐invasive methods available for studying the human placenta, tissue collected from term deliveries was used for the quantification of mRNA, protein, and cell types. The results from term tissue may not be generalizable to other gestational timepoints. Additionally, immunofluorescence is a semi‐quantitative and subjective method. We attempted to control for the subjective nature of the technique by defining an objective set of parameters to be followed before commencing cell counting, as well as having one researcher conduct cell counting, thus decreasing interobserver variability. Blinding of the images also reduced any potential researcher bias. Another possible risk of immunofluorescence is bleed‐through of the secondary antibodies, potentially resulting in false positive staining. To mitigate this risk, fluorophores with distinct spectra were selected. In performing immunofluorescence, we used a previously published protocol enabling the utilization of two primary antibodies from the same species (Tzaneva et al., 2014; Tzaneva & Perry, 2016). While the use of two primary antibodies from the same species could potentially lead to cross‐reactions, by incubating the first round of secondary antibodies overnight, they were allowed to saturate the binding sites available for the mouse anti‐CD68 antibody. Following this, the sections were thoroughly washed to remove any unbound goat anti‐mouse secondary antibody. This mitigates the risk of binding between the mouse anti‐CD68 primary antibody and goat anti‐mouse Alexa 649 antibody, and between the mouse anti‐VEGF primary antibody and the goat anti‐mouse Alexa 488 secondary antibody. The staining patterns of CD68 and VEGF were assessed and found to have different staining patterns (Figure 8).

Our study also possesses multiple strengths, including the use of accelerometry as a validated objective measure of PA; measuring PA at multiple time points throughout gestation; controlling for potential confounding factors such as maternal age, BMI status, and gestational age at delivery; and having a standardized protocol for placenta collection. Additionally, we use a tissue homogenate of multiple placental locations to account for tissue heterogeneity throughout the placenta.

## 5 CONCLUSIONS AND FUTURE DIRECTIONS

In conclusion, VEGF was seen to be expressed in M2a, M2b, and M2c phenotypes. PA was observed to be positively correlated with M2a and M2c HBCs as a proportion of the total in term placenta, with a large effect size seen relative to PA levels mid‐gestation (*r* > 0.5), and a moderate effect size seen in late gestation (*r* > 0.3). While flow cytometric analyses were not possible in this pilot study due to a lack of fresh tissue, these results provide a strong basis for future research to examine polarization differences using fluorescence‐activated cell sorting. Placental CD206 protein expression was lower in individuals who were physically active throughout pregnancy compared to ones who were inactive. Placental CD206 mRNA levels were not significantly different between active and inactive individuals, suggesting a potential PA‐mediated downregulation of CD206 during translation. The cause of this hypothesized protein downregulation and potential downstream consequences should be explored in further studies. Future research should also ensure that the proportions of cells are examined in conjunction with absolute numbers to avoid overlooking potentially significant discoveries. Finally, the amount of VEGF secreted from distinct HBC subtypes should be investigated in healthy and complicated pregnancies to determine if changes in polarization may affect the amount of VEGF secreted.

## AUTHOR CONTRIBUTIONS

ADG, VT, and KBA contributed to study conception, design and data analysis. ADG, CE, LT, and VT carried out primary data collection and interpretation. ADG and CE performed data analysis. ADG wrote the manuscript with KBA as the corresponding author. All authors revised, edited, and approved the final version of the manuscript. All authors revised and edited the manuscript and have approved the final version.

## FUNDING INFORMATION

This study was supported by grants from the Canadian Institutes of Health Research (MOP 142298) and the Natural Sciences and Engineering Research Council (RGPIN‐2017‐05457) awarded to KBA.

## CONFLICT OF INTEREST STATEMENT

The authors have no conflict of interest to declare.

## ETHICAL STATEMENT

The authors confirm that the ethical policies of the journal, as noted on the journal's author guidelines page, have been adhered to and the appropriate ethical review committee approval has been received. The PLACENTA study was approved by the Research Ethics Board (REB) of the University of Ottawa (file number: H11‐15‐29) and conformed with all aspects of the Declaration of Helsinki.

## Data Availability

The data that support the findings of this study are available from the corresponding author, KBA, upon reasonable request.
